# A Multi-Pump Magnetohydrodynamics Lab-On-A-Chip Device for Automated Flow Control and Analyte Delivery

**DOI:** 10.3390/s20174909

**Published:** 2020-08-31

**Authors:** Rafael M. Cardoso, Robson O. dos Santos, Rodrigo A. A. Munoz, Carlos D. Garcia, Lucas Blanes

**Affiliations:** 1 Institute of Chemistry, Federal University of Uberlândia, Uberlândia 38400-000, Brazil; rcardosoq@gmail.com (R.M.C.); munoz@ufu.br (R.A.A.M.); 2 Laboratory for Applied Science and Technology in Health, Carlos Chagas Institute, Oswaldo Cruz Foundation (Fiocruz), Curitiba 81350-010, Brazil; robsonos@outlook.com; 3 Department of Chemistry, Clemson University, Clemson, SC 29634, USA

**Keywords:** MHD, flow analysis system, magnetohydrodynamics, lab-on-a-chip

## Abstract

This article shows the development of a computer-controlled lab-on-a-chip device with three magnetohydrodynamic (MHD) pumps and a pneumatic valve. The chip was made of a stack of layers of polymethylmethacrylate (PMMA), cut using a laser engraver and thermally bonded. The MHD pumps were built using permanent magnets (neodymium) and platinum electrodes, all of them controlled by an Arduino board and a set of relays. The implemented pumps were able to drive solutions in the open channels with a flow rate that increased proportionally with the channel width and applied voltage. To address the characteristic low pressures generated by this kind of pump, all channels were interconnected. Because the electrodes were immersed in the electrolyte, causing electrolysis and pH variations, the composition and ionic strength of the electrolyte solution were controlled. Additionally, side structures for releasing bubbles were integrated. With this multi-pump and valve solution, the device was used to demonstrate the possibility of performing an injection sequence in a system that resembles a traditional flow injection analysis system. Ultimately, the results demonstrate the possibility of performing injection sequences using an array of MHD pumps that can perform fluid handling in the 0–5 µL s^−1^ range.

## 1. Introduction

Following the rise of microfluidic studies, there has been an increase in the number of research projects involving lab-on-a-chip (LOC) devices, especially those aiming to create analytical approaches that are reliable, inexpensive, portable, and accessible [1]. Functional LOC devices require accurate fluid manipulation to achieve filtration, mixing, or separation of components [2]. This is usually accomplished by integrating one or more pumps. Among other approaches reported in the literature [3,4,5,6], the most common micropumps rely on the use of electro-osmosis [7] or pneumatic actuation [8]. Although these alternatives provide robust ways to displace solutions, their operation requires either a network of gas lines and valves or high-voltage power supplies and a configuration that can support electro-osmosis.

Magnetohydrodynamic (MHD) pumps have become a convenient alternative to the development of LOC devices, showing multiple advantages such as simple and compact arrangements, low cost, possibility of flow inversion, reduced noise operation, high efficiency, short transient time, and minimal maintenance [9,10,11,12]. These non-mechanical pumps work by combining an electric field and an orthogonal magnetic field, resulting in a Lorentz force with the flow direction defined by the current and magnetic field vectors.

Direct-current MHD micropumps were first reported in the literature in 2000 by Jang and Lee [13], who used silicon channels, an NdFeB permanent magnet (0.44 T), and a saline solution to achieve flow rates up to 63 µL min^−1^ (head pressures of 18 mm of water at 38 mA). In 2001, Gao et al. demonstrated the capability of MHD to control the delivery of a substance through flow channels [14], providing several additional theoretical and practical considerations. Later, several reports explored MHD as a fluidic propeller using direct current for MHD micropumps in different applications. Among these, it is worth mentioning co-fired ceramic tapes with the electrodes printed on the ceramic substrate, which were used to propel mercury slugs, saline solution, and deionized water [15,16,17].

For stirring purposes, Bau et al. developed an MHD stirrer that exhibits chaotic advection with individual electrodes positioned along its opposing walls [18]. Fritsch et al. pioneered several MHD-based devices for fluid handling, such as an MHD-driven flow using redox species [19,20,21]. Recent advances of this research group have focused on lab-on-a-chip devices that are able to handle fluids without sidewall channels, without bubble generation, and without the need to use redox species [22,23]. One of the advantages of adding redox species to MHD-based systems is the possibility of avoiding bubble generation on electrodes and their consequent degradation, as the redox species serves as a sacrificial element. Another strategy to avoid bubbles in MHD pumps is adding side bubble-release channels, in which the electrodes are inserted [24,25]. MHD pumps have also been used in resonance environments [24,25,26] and in multi-pump solutions capable of delivering a sample to an integrated electro-immunoassay [27].

Despite these advantages, the practical applicability of MHD pumps has been limited due to difficulties associated with trying to run these devices long enough to perform a series of analytical operations (injections, mixing, washing, etc.).

Aiming to address these shortcomings, this report describes a simple device that incorporates three pumps, one valve, three reservoirs (for running electrolyte, sample, and waste), and a chamber to perform mixing. This same chamber is also used as a detection window, where color changes are measured. The flow control and analyte delivery are performed in a manner that resembles a pumpless flow injection analysis system, which represents a novel strategy to perform sequential injections in the device. In order to rationally optimize the operation of the device, the effects of the architecture, applied potential, and electrolyte composition were investigated.

## 2. Materials and Methods

### 2.1. Chemicals

Anhydrous sodium monobasic phosphate (99%) was purchased from Fisher Scientific (Fair Lawn, NJ, USA), citric acid (99%) was purchased from Aldrich (Cincinnati, MO, USA), methylene blue trihydrate was purchased from MP Biomedicals (Solon, OH, USA), potassium chloride (99%) was purchased from EM Science (St. Louis, NJ, USA), and boric acid (99.9%) was purchased from Mallinckrodt Chemicals (Phillipsburg, NJ, USA). All solutions were prepared using ultrapure water with 18 MΩ cm of resistivity (NANOpure^®^ Dlamond^TM^ from Barnstead; Dubuque, IA, USA). Standard-grade PMMA plates of different sizes (150 mm × 70 mm× 1.52 mm and 150 mm × 70 mm × 3.17 mm) were purchased from Gravograph (Duluth, GA, USA). Platinum wires were purchased from Aldrich (Cincinnati, MO, USA), and neodymium bar N52 magnets (60 mm × 10 mm × 5 mm) were purchased from DIYMAG, China (model # HLMAG01, via Amazon).

### 2.2. Electronics and Hardware

A power supply with a 0–30 VDC and 0–10 A output was used to power the MHD pumps. The device was controlled using an Arduino Mega 2560 development board, with 60 input/output pins exposed. The interface between the Arduino board and the device was made with a four-channel relay module. The hardware used for the detection was a USB 2.0 digital microscope camera, which was used to record time-lapse videos with a resolution of 640 pixels × 480 pixels. The electronic parts used in the multi-pump solution are listed in the Supplementary Information section.

### 2.3. Apparatus

The devices were built using a CO_2_ laser engraver, Mini24 (30 W), from Epilog Laser Systems (Golden, CO, USA). More details about the laser engraver can be found in previous reports [28,29]. A commercial thermal transfer press, Stahls’ Hotronix model STX11, was used to seal the PMMA plates. An Orion^®^ pH meter was purchased from Thermo (Waltham, MA, USA).

### 2.4. Construction and Operation of the Single-Pump MHD Chip

**Figure 1:**
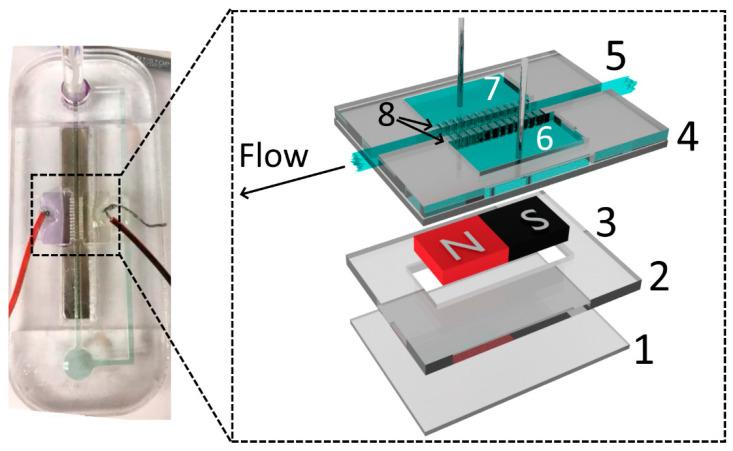
Picture of a single-pump magnetohydrodynamic (MHD) chip. Inset shows the schematic diagram of an MHD pump: (**1** and **2**) PMMA magnet holders, (**3**) N52 magnet, (**4**) PMMA chip, (**5**) main channel (1.53 mm width), (**6** and **7**) side bubble release reservoirs with electrodes, (**8**) grooves used to connect the electrolyte from the side reservoirs to the main channel.

The devices were designed using CorelDRAW^®^ Graphics Suite X6, and the layers of PMMA were cut using the laser engraver. The main channels were cut using 100% power and at 10% speed (maximum linear speed of 1650 mm/s) on the 150 mm × 70 mm × 3.17 mm PMMA plates. The engraving procedure was repeated twice. These settings were selected to completely cut out the acrylic parts. To seal the layers, PMMA pieces were heated to a temperature of 240 °C and pressed together for 6 min using the heat press. To avoid warping the acrylic pieces, the heating function was turned off and the device was maintained in the press until reaching room temperature (approximately 1 h). The side reservoirs (for bubble release, 15.3 mm × 7.8 mm) were connected to the main channels via small groves, also fabricated with the laser engraver at 40% speed and 100% power and then sealed facing downwards. The electrodes (5 to 30 V applied) were placed in those side reservoirs using 2 mm PMMA holders, enhancing the mechanical resistance. Two extra layers of PMMA were fixed at the bottom of the device to keep the magnets in place. Figure 1 shows a schematic representation and a picture of a device featuring a single MHD pump.

This single-pump MHD chip was developed to optimize parameters such as channel width, electrolyte composition and concentration, and pH. The MHD flow (μL s^−1^) was calculated by pipetting 10 μL of methylene blue dye (0.2 g L^−1^) on the main channel and recording the time spent by the dye to move through a fixed distance. This procedure was chosen due to its simplicity, and despite the limitations of this method, it can concisely measure the variations between different potentials/electrolytes in the flow observed.

### 2.5. Construction and Operation of the Multi-Pump MHD Chip

**Figure 2:**
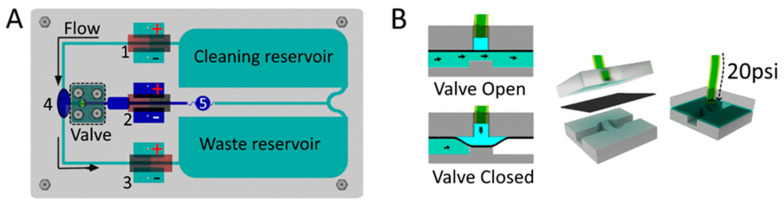
MHD chip with a multi-pump network: (**A**) Superior view: 1, 2, and 3 are the MHD pumps; 4 is the detection cell and detection point (RGB); and 5 is the analyte/electrolyte mixing barrier. (**B**) Lateral view of the valve: the valve is normally open, but when pressure (20 psi) is applied on the liquid connected to the polydimethylsiloxane (PDMS) membrane layer, the valve closes.

Figure 2A shows the main components present in the device. The multi-pump network consists of three MHD pumps (marked as pumps 1, 2, and 3), one valve, two electrolyte reservoirs, and a detection window (4) to perform the mixing and reading of color intensities. To perform the automation of the pumps and valves, a computer-controlled system was constructed. The system used an Arduino board to control four relays: three of them to control the MHD pumps and one to control the valve (see Section 2.6 and Section 2.7). The MHD injection pump (pump 2 in Figure 2A) created a flow towards the T-junction reservoir. The MHD circulating pumps (pumps 1 and 3 in Figure 2A) were connected in series to create a flow moving in the anticlockwise direction. It is worth mentioning that the polarity of the Pt electrodes can be inverted to change the flow direction in each pump. The multi-pump network was developed to work as a fully connected network, providing hydrodynamic paths where the reservoirs at the extremity of the chip were filled with electrolyte to sustain the cleaning step after passing through the MHD pump.

For the construction of the multi-pump MHD chip, a 162 mm × 92 mm × 3.17 mm PMMA plate was used, with a 1.53 mm wide channel, two main electrolyte reservoirs, six smaller reservoirs for side bubble release (two for each MHD pump), and an oval-shaped detection cell along with its channel. The applied laser engraving procedure is described in Section 2.4 Since the vector mode leaves a cross-sectional hole in the acrylic, an uncut part was purposely left to prevent the acrylic cut plate from falling apart, supporting the structure containing the channels and reservoirs. After that, a PMMA plate with 1.53 mm thickness and the same dimensions was sealed using the same procedure as described in Section 2.4 After sealing the bottom acrylic layer, a small part (only used to support the structure) was removed with a drill bit, ensuring that the channels were connected. The magnets were placed in the pocket holes, then screwed against the chip plate, making a sandwich-type chip with four layers. The two first layers were thermally sealed to each other, in which the first one presented the channels and the second was a flat PMMA board used to seal the channels. The third layer was the magnet aligner layer, and the bottom layer was used to hold the magnet, fixed using M3 screws and nuts. The Pt electrodes were placed at the reservoirs to obtain a current that would be affected by the magnetic field, generating the flow. The entire chip was filled with 10 mL of electrolyte. An amount of 8.6 mL was used to fill the waste and cleaning reservoirs and the channels that connected them to MHD pumps 1 and 3. An additional 1.4 mL of the electrolyte containing the blue dye/sample was added in the middle of MHD pump 2 to fill that entire area, which was used as a sample reservoir. This procedure was made with the valve closed (Figure 2B). Reservoir 5 in Figure 2A was created to avoid the contamination of the cleaning and waste reservoirs with the sample. When the valve was opened and the MHD pumps were turned on, the sample moved through the detection chamber.

### 2.6. Valve Construction and Operation

A pneumatic single valve was created to control the injections using a flexible polydimethylsiloxane (PDMS) layer in between the acrylic chip part and the bottom of the channel (see Figure 2B). The layers were screwed together to avoid pressure leaking, and the valve inlet was connected to a CO_2_ cylinder using 20 psi of pressure to close the valve. The pneumatic valve was operated electronically via a solenoid (LHDA1221111H, Lee Co.; Essex, CT, USA) interfaced with the Arduino board (see Supplementary Information). The valve was presented in a normally open configuration and was activated between the programmed injections. The PMMA fluidic wafer (designed to fit the PDMS layer) was carved with the laser cutter and engraving machine, using the raster mode, at 50% speed and using 20% power for four times, until a curved space was obtained. Since the PDMS layer is slightly permeable to gases (and could lead to the formation of bubbles), the tube connected to the valve that injects the air was filled with 1.5 mL of water.

### 2.7. Firmware, Software, and Operation

Both firmware and software were programmed using Visual Studio Code (http://code.visualstudio.com). The firmware for the Arduino board (http://store.arduino.cc/usa) was developed in C++ using the PlatformIO tool for embedded systems (http://platformio.org), and the software for the apparatus was developed in JavaScript through the Electron framework (http://electronjs.org). In order to test different scenarios for the flow, the software was divided into six routines, which were executed in a sequential loop. The control of the chip was then achieved by manipulating the time in which each relay was turned on or off, for a defined time in each sequence controllable by the user (see Supplementary Information). The images were collected using a digital microscope camera (Plugable Technologies, Redmond, WA) in time-lapse mode every 2 s. The mean values of the sum of the vectors R, G, and B were obtained and processed using ImageJ software (https://imagej.nih.gov/ij/).

## 3. Results and Discussion

### 3.1. MHD Chip Optimization

**Figure 3:**
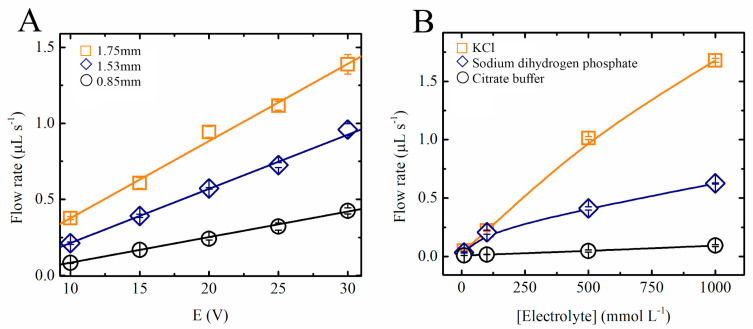
Study of the fluid movement on a single MHD pump (see Figure 1): (**A**) Flow rate as a function of electrical potentials applied for different channel widths using 0.5 mol L^−1^ NaH_2_PO_4_ as electrolyte. (**B**) Flow rate as a function of the concentration of the electrolyte for different ionic compositions using a fixed electrical potential and channel width of 15 V and 1.53 mm, respectively.

The MHD pump shown in Figure 1 was developed to optimize the system parameters. One of the common drawbacks of MHD pumps is that they are only able to generate small pressure differences [13,24], requiring the system to be connected in a closed loop. For that reason, and considering that the described system was intended to perform multiple analytical operations, the effect of the most significant experimental variables on the proposed MHD pumps was first investigated. Fritsch’s research group [21] demonstrated an MHD channel-less experimental setup, with flow in a width path of around 250 µm mounted on a glass and PDMS substrate. Homsy et al. demonstrated a chip with channels using a combination of Pyrex wafer and PDMS, reaching 150 µm width and 75 µm depth [24]. The criterion for choosing wider channels and a high current throughput involves evaluating the possibility of using an MHD pump on a microfluidic system. Figure 3A shows a summary of the results obtained with three different chips, designed to investigate the effect of the channel widths (0.85, 1.54, and 1.75 mm) and applied potentials (10, 15, 20, 25, and 30 V) on the flow rate. These experiments were performed using 0.5 mol L^−1^ NaH_2_PO_4_ as the background electrolyte and measuring the time required for a drop of methylene blue (10 µL/0.2 g L^−1^) solution to move through the channel (69 mm in length). In line with previous reports, our results show that the linear velocity was proportional to the applied potential (R^2^ > 0.99) and that wider channels led to higher flow rates (Figure 3A). These trends have been attributed to the linear dependence between the flow rate and the current in the pump [4].

It has been established that most considerations linked to MHD pumps are applicable to fluids with a given electrical conductivity, which in turn affect the induced current density [11] and thus the flow rate. Because typical applications for these pumps require the use of different experimental conditions, the linear velocity and flow rate of the proposed pumps was investigated as a function of the type of electrolyte used. Figure 3B shows that not only the concentration of the electrolyte but also the mobility of the ions had a significant effect on the linear velocity in the MHD pumps. As expected, electrolytes composed of citrate or phosphate (H_2_PO_4_^−^) displayed lower linear flow velocities than those containing KCl.

It is also important to mention that although solutions containing 0.1 mol L^−1^ KCl provided higher conductivity and higher flow rates when compared with those containing NaH_2_PO_4_, the redox chemistry of the ions also led to significant increases in the pH of the running electrolyte, leading to the formation of bubbles, and to the production of Cl_2_ (pH variation measurements not shown). On the other hand, when phosphate solutions (H_2_PO_4_^−^ at 0.5 mol.L^−1^) were selected, no significant changes in the pH values were observed (30 min operation). The presence of KCl in the solution caused the pH variation to happen faster due to the higher activity of K^+^ and Cl^−^ ions, leading to higher current densities and considerable higher flow rates. Additionally, the fact that the use of MHD pumps is compatible with a range of phosphate-buffered solutions indicates that the system could be applied to conduct enzyme-based assays in the future as most enzymes require neutral media (pH 7) to keep their maximum efficiency. Different enzymes can be easily immobilized within the microfluidic channel.

### 3.2. MHD Multi-Pump Chip with RGB Detection

**Figure 4:**
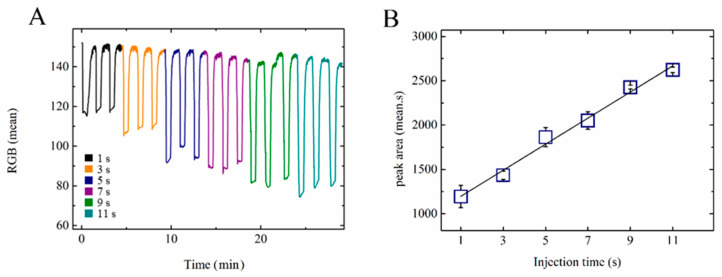
(**A**) RGB detection (camera + ImageJ software) of methylene blue dye, 0.2 g L^−1^, in NaH_2_PO_4_, 0.5 mol L^−1^, pH 4.3, 15 V, using different injection times. Reading and cleaning times of 20 and 60 s, respectively. (**B**) Illustration of the correlation between the injection time and RGB mean.

The automated multi-pump network was tested in successive and programmable injections, integrating three MHD pumps and one valve in a 10 mL chip, as shown in Figure 2. The experiment was made using a 0.5 mol L^−1^ NaH_2_PO_4_ buffer at pH 4.3. In order to understand the injection system, a methylene blue solution was injected under different time conditions (see Figure 4). As described in Section 2.5, 1.4 mL of methylene blue (0.2 g L^−1^) was injected using MHD pump 2 (see Figure 2). The T-junction reservoir that connected the electrolyte path and analyte channels was positioned right after the pneumatic valve and featured an oval shape to increase the homogenization of the analyte–electrolyte mixture. This spot was the detection chamber, where the camera was positioned to measure the color intensities. It was observed that when the size of the detection chamber reservoir was too small, shorter injection times were enough to saturate the reading spot area under the specific electrolyte and voltage conditions. The dimension of the detection chamber was defined based on a condition in which the concentration of methylene blue changed as a function of the injection time (1–11 s), which was the interval in which the valve and the injection pump were turned on. The signal obtained after treatment in ImageJ was the RGB mean (see Supplementary Information—Video S1).

During the cleaning step, the valve was kept closed, while MHD pumps 1 and 3 were turned on. In this configuration, MHD pump 1 pushed the liquid towards the detection chamber, and at the same time, pump 3 sucked the liquid in the direction of the waste reservoir. During the injection, the valve was opened for a few seconds (see Figure 4) and MHD pump 2 was turned on. After injection, the valve was closed for 20 s for the reading step, before starting a new cycle. The RGB mean intensity variation along with the injections led to transient peaks, as shown in Figure 4. It is possible to observe in Figure 4B that there was a linear relationship between the color intensity and the injection time (1 to 11 s). Further increases in the injection time led to cross-contamination due to the recirculation of the solution—an issue that highlights the importance of considering the total volume of the device. We also conclude that the use of a computer-controlled system helped to obtain more reliable injections and therefore better analytical results. 

## 4. Conclusions

This communication shows the design of an interconnected LOC device with MHD pumps and reservoirs that performs fluid handling in the 0–1.5 µL·s^−1^ range. These values can be achieved by selecting potentials <30 V and electrolytes with concentrations lower than 1 mol·L^−1^. Although these pumps do not generate much head pressure, the liquid movement occurs accordingly, with higher flow rates occurring in wider channels. Of course, this is a limitation of the method since the liquid movement will be reduced if micro channels are used. Despite that, an entire LOC device with integrating channels (total volume = 10 mL), reservoirs, and a pneumatic valve was created, automatically allowing sequential injections (1–11 s range) for 30 min. The results also show that by considering the volumes of the reservoirs (in relation to the flow rates), full devices can be designed to run autonomously, without significant cross-contamination. Additionally, characteristic issues related to cross-contamination or electrolysis effects in LOC with MHD pumps can be minimized if they are properly controlled using a computer-controlled system.

## Supplementary Materials

The following are available online at https://www.mdpi.com/1424-8220/20/17/4909/s1, Figure S1: Schematic representation of the electronic hardware controller; Figure S2: In-house-developed software to control the chip flow. The routines are run in order from 1 to 6. Each routine is composed of 3 activation sequences: inject, run, and clean. For each sequence, it is possible to select the channels to the corresponding relays (checkboxes) and time for the sequence in seconds (input field on the right). The sequences are run in order (inject, run, and clean). The functions for the relays are specified as follows (see Figure S1 for details): 1—MHD injection pump; 2—MHD cleaning pump; 3—disconnected; 4—pneumatic injection valve. Figure S3: Image obtained from the USB digital microscope, using ImageJ^®^ software, showing the RGB detection at the T-junction chip. Figure S4: Picture of the multi-pump apparatus used for methylene blue injections and D-glucose assay kit. Video S1: Capture of a sequence of dye injections, followed by the rinsing step.
