# Supplementary material for: A Multi-Pump Magnetohydrodynamics Lab-On-A-Chip Device for Automated Flow Control and Analyte Delivery
Source: Sensors (Basel). 2020 Aug 31;20(17):4909. doi: 10.3390/s20174909 (PMC7506898; doi:10.3390/s20174909)
Supplement: Supplementary file 1 [file sensors-20-04909-s001.pdf]

sensors

MDPI

Supplementary information

A Multi-pump Magnetohydrodynamics Lab on a Chip Device for Automated Flow Control and Analyte Delivery

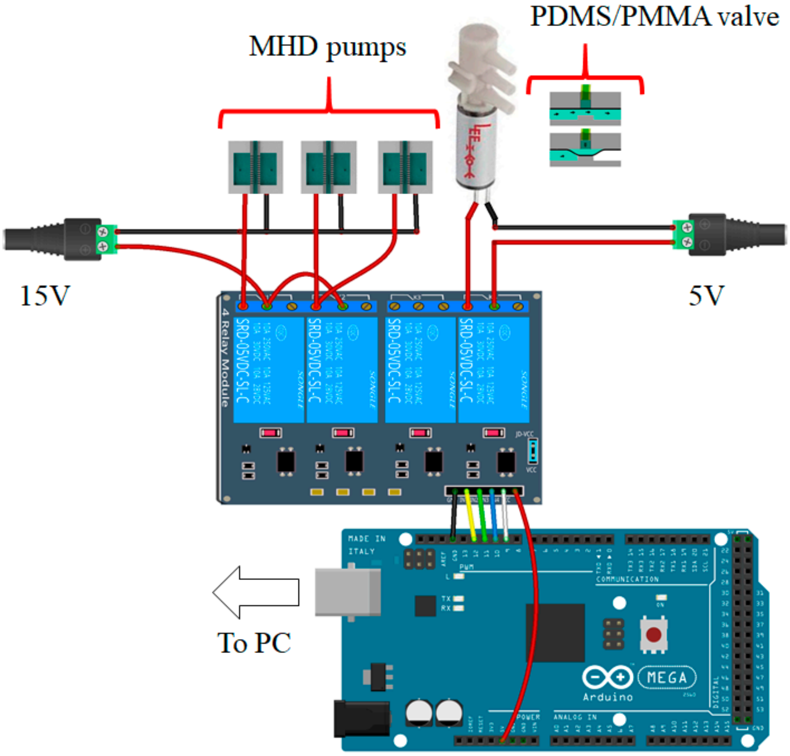

Figure. S1. Schematic representation of the electronic hardware controller.

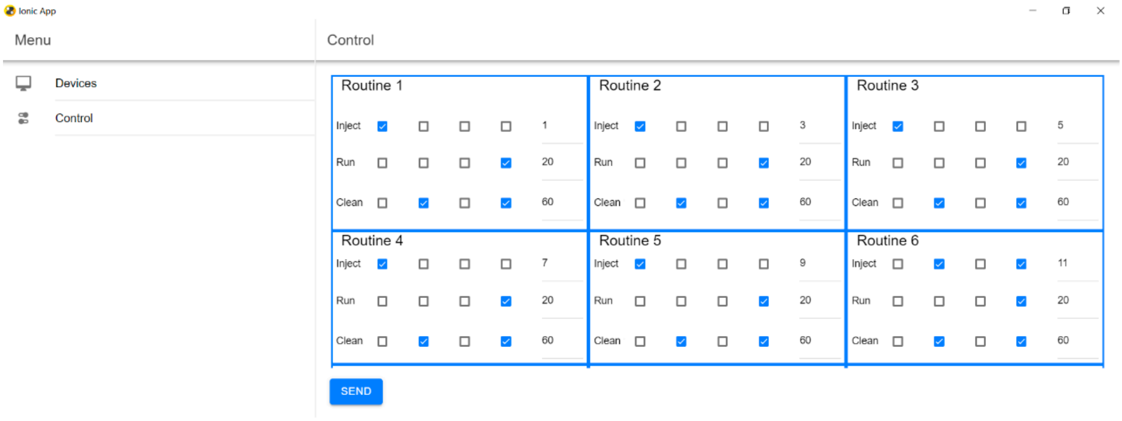

Figure S2. In-house developed software to control the chip flow. The routines are run in order from 1 to 6. Each routine is composed of 3 activation sequences: inject, run and clean. For each sequence, it is possible to select the channels to corresponding relays (checkboxes) and time for the sequence in seconds (input field on the right). The sequences are run in order (inject, run and then clean). The function for the relays is specified as follows (See Figure. S1 for

details): 1 – MHD injection pump; 2 – MHD cleaning pump; 3 – Disconnected; 4 – pneumatic injection valve.

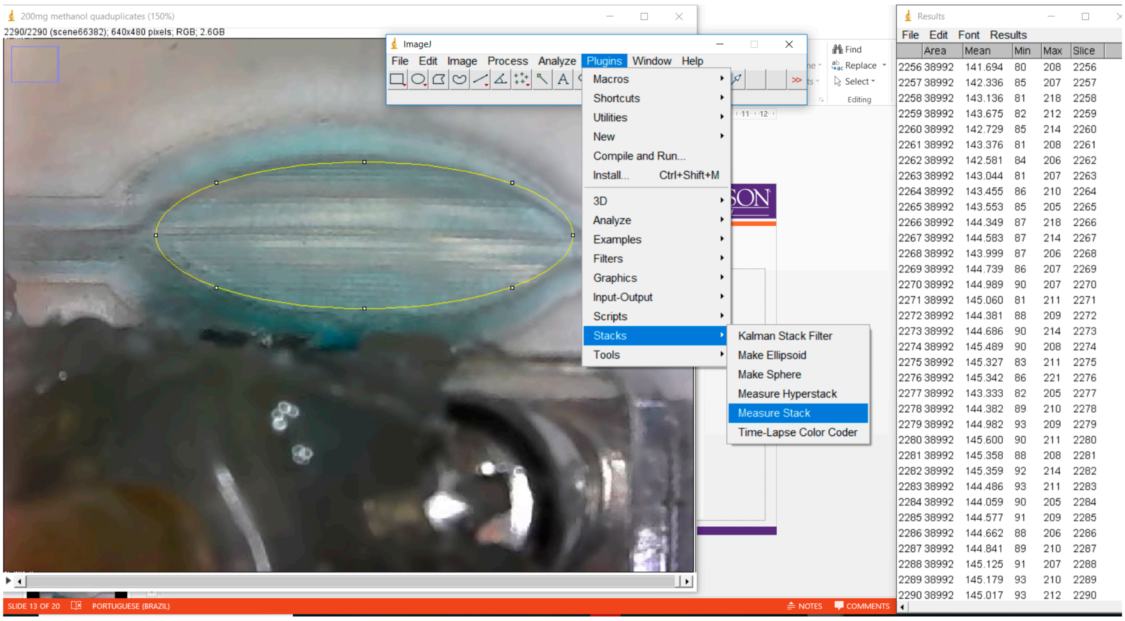

Figure S3. Image obtained from the USB Digital Microscope, using the Image J® software, showing the RGB detection at the T-junction chip.

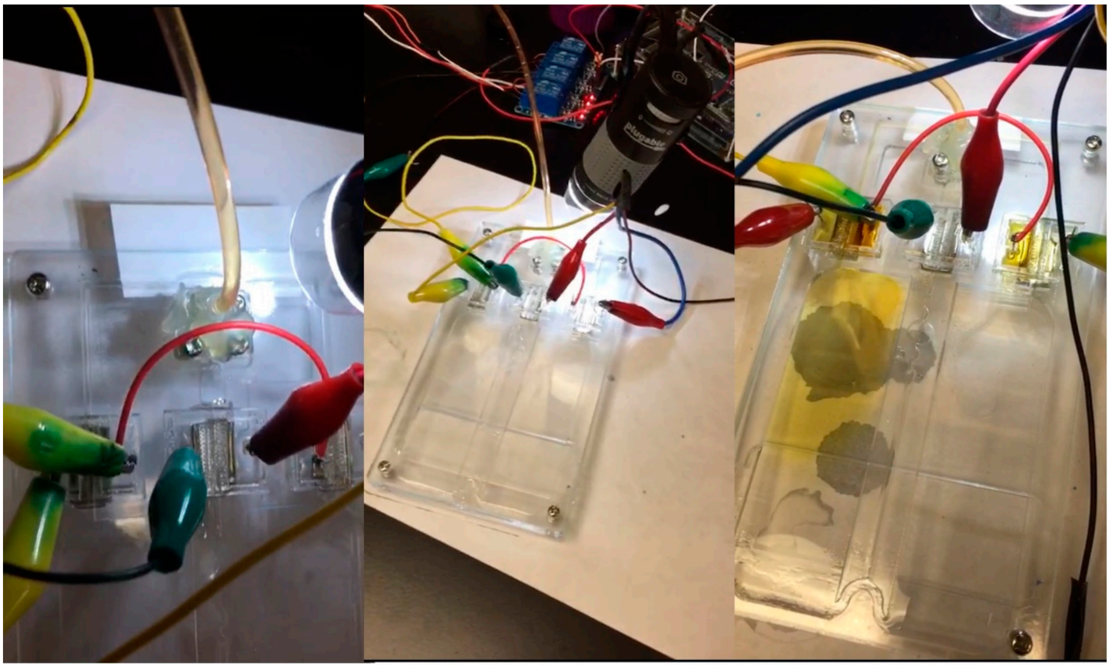

Figure S4. Picture of the multi-pump apparatus used for Methylene Blue injections and D-Glucose assay kit.

Table S1. List of hardware used in this paper with manufacturer and location.

Model	Manufacturer	Location
Power supply CSI3010SW	Circuit-specialists	AZ, USA
Controller board Arduino	Arduino Atmel	AZ, USA
Relay module SRD-05VDC-SL-C	JBtek	Surrey, Canada
USB 2.0 Digital Microscope	Plugable Technologies	Redmond, WA

© 2020 by the authors. Submitted for possible open access publication under the terms and conditions of the Creative Commons Attribution (CC BY) license (http://creativecommons.org/licenses/by/4.0/).
